# Editorial: AI-driven advances in immunology and immune-mediated disorders

**DOI:** 10.3389/fimmu.2026.1924665

**Published:** 2026-09-03

**Authors:** Diego Ulisse Pizzagalli, Giovanni Porta, Inge M. N. Wortel

**Affiliations:** 1 Theodor Kocher Institute, Faculty of Medicine, University of Bern, Bern, Switzerland; 2 MeDiTech Institute, Department of Innovative Technologies, University of Applied Sciences and Arts of Southern Switzerland (SUPSI), Lugano, Switzerland; 3 Center for Genomic Medicine, Department of Medicine and Surgery, Università degli Studi dell’Insubria, Varese, Italy; 4 Institute for Computing and Information Sciences, Radboud University, Nijmegen, Netherlands

**Keywords:** AI-driven immunology, bioimage analysis, biomedical information retrieval, computational immunology, systems immunology

Current researchers have unprecedented access to data generated from various sources, such as high-throughput sequencing, mass spectrometry, advanced imaging technologies and clinical records. Going hand in hand with rapid advancements in artificial intelligence methods, this provides a range of new opportunities and challenges to study the intricate mechanisms of the immune system and finally improving the outcome of immune-mediated disorders. In this editorial, we highlight how the contributions in this research topic reflect ongoing advancements in immunology and immune-mediated disorders that are enabled by artificial intelligence (**Figure 1**).

**Figure 1:**
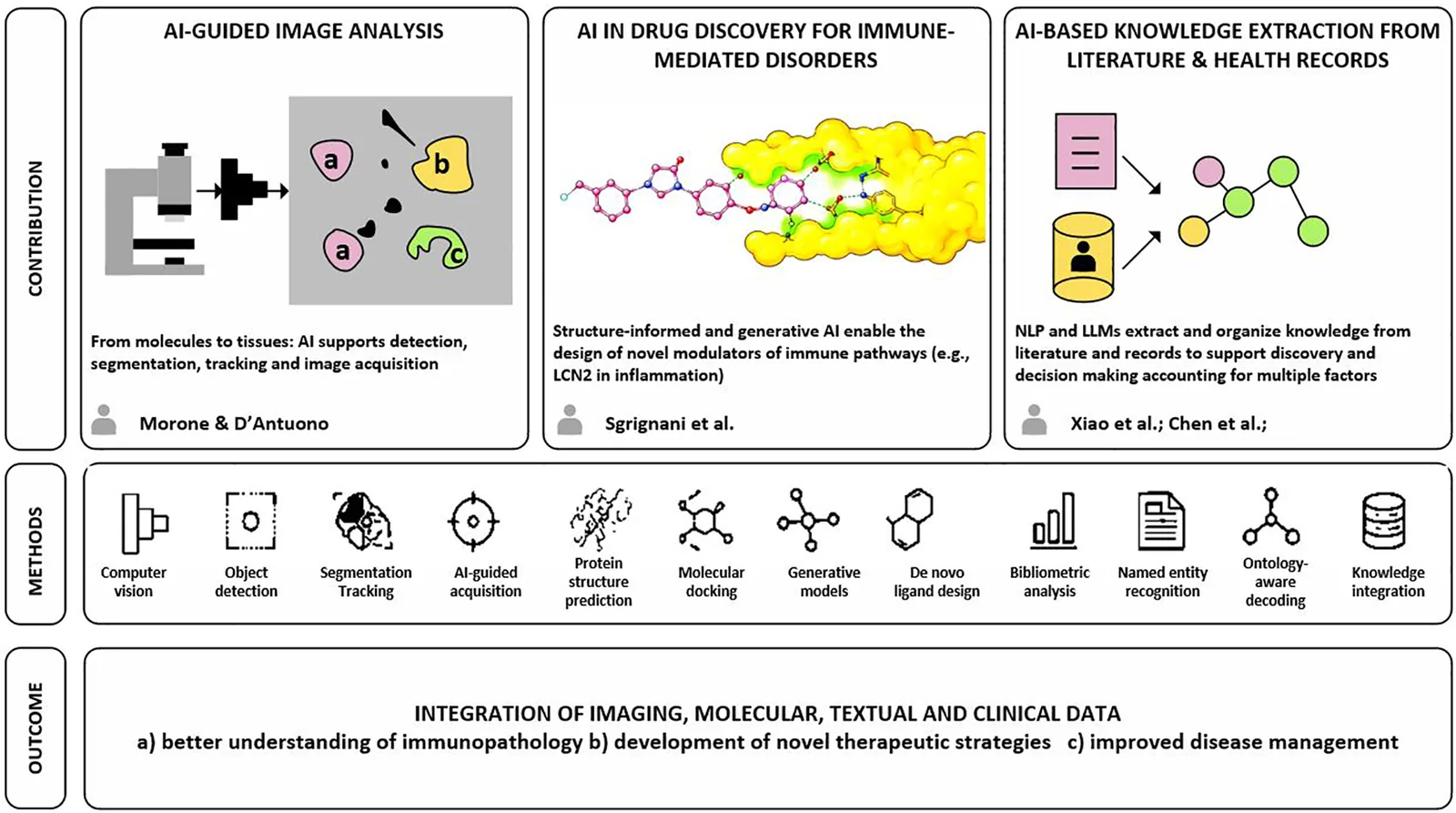
Diagram summarizing AI contributions to immunology presented in this research topic, showing three columns: AI-guided image analysis, AI in drug discovery for immune-mediated disorders, and AI-based knowledge extraction from literature and health records. Each section lists relevant methods such as computer vision, molecular docking, and natural language processing. Outcomes include improved immunopathology understanding, novel therapeutic strategies, and better disease management.

## AI in imaging the immune system

One of the earliest areas where modern AI approaches became clearly competitive was in computer vision, benefiting from well-defined benchmarks and large labeled datasets.

These developments have been translated to immunology enabling a broad spectrum of applications as - alongside more traditional approaches such as flow cytometry - both static and imaging data play an increasing role in immunological research across different scales. At the molecular scale, imaging of the immunological synapse has revealed the nanoscale clustering of molecules involved in T cell activation. At the cellular scale, intravital and live-cell imaging have visualized immune cells as they detect, migrate toward, and eliminate infected cells. At the tissue scale, multiplex immunohistochemistry and spatial transcriptomics have become key tools for assessing spatial relationships between immune cells and cancer, helping to understand immune infiltration in the tumor microenvironment and its potential link to therapy responses.

In their review, Morone and D’Antuono discuss the role various types of imaging have played in immunology and virology and outline the different ways in which AI can assist this process—from traditional computer vision tasks such as detecting, segmenting, and tracking objects, to AI-guided image acquisition. They discuss limitations and sketch potential applications of AI across the (imaging) research cycle.

## AI in drug discovery for immune targets

Aside from advances in computer vision, immunology is largely benefiting of AI in structural biology. One key breakthrough has been the development of AlphaFold and related models in protein structure prediction and molecular modelling. AI-guided workflows for drug discovery now allow the design of *de novo* ligands targeting specific proteins across a range of biomedical fields.

This is especially relevant to drugging immune signalling pathways that include a large number of well-characterized but therapeutically challenging targets such as cytokines, receptors, and acute-phase proteins. In this field, AI-guided drug discovery is of interest to help design tailored and specific modulators of immune responses. Sgrignani et al. show how AI-guided drug discovery can be applied to target components of the inflammatory response, such as LCN2, a glycoprotein upregulated in the acute phase of infection and inflammation.

Their work represents the recent shift from screening-based drug discovery toward structure-informed design frameworks powered by advances in protein modelling and deep learning. In the context of immune-related disorders, this shift may facilitate the development of drugs targeting immunological pathways that have traditionally been difficult to drug. As the pace of AI-assisted drug development accelerates, robust biological validation standards are becoming increasingly important and remain an active frontier of research. Beyond ensuring drug safety, validation is essential to address the substantial gap between in silico predictions and *in vivo* drug activity. Without rigorous experimental validation, there is a risk of creating a closed-loop system in which increasingly large numbers of theoretical candidates are generated and propagated through the literature despite lacking functional activity *in vivo*.

## AI in knowledge extraction from immunology literature and health records

Alongside advances in imaging and molecular modelling, artificial intelligence is increasingly being applied to extract and organize knowledge from textual sources. Natural language processing and large language models now enable the identification of relationships across biomedical concepts. These approaches are becoming an important complement to both experimental research and clinical practice, helping transform unstructured scientific text into structured knowledge that can support discovery and decision-making.

In this Research Topic, Xiao et al. provide a comprehensive bibliometric analysis of AI applications in allergy and immunology, mapping the evolution of the field and identifying emerging research directions. Their analysis highlights the growing role of AI across diverse areas, including disease prediction, diagnostics, drug development, and clinical decision support. Complementing this work, Chen et al. address one of the core challenges of biomedical text mining by developing a domain-specific named entity recognition framework for immunology. Their approach improves the identification of complex immunological entities from scientific literature by combining structured representations with ontology-aware decoding, achieving more accurate extraction of immune-mediated disease dynamics.

Together, these studies demonstrate how AI can accelerate knowledge discovery by both synthesizing scientific evidence and extracting structured information from biomedical publications. As the volume of immunological literature continues to grow, such methods will become increasingly important for supporting literature curation, biomarker discovery, and the translation of accumulated knowledge into clinical and research applications. They also complement the growing use of AI to integrate textual information with clinical and molecular data, enabling applications ranging from the identification of patients at risk of autoimmune diseases to the development of quantitative measures of immune health.

## Challenges

The application of AI in immunology has the potential to shed light on intricate networks of molecular and cellular interactions, as well as to characterize the evolution of immune-mediated disorders over time. Although AI methods are increasingly fast, accurate, and capable of detecting the complex patterns described above, transparency and interpretability of their results remain important considerations for their use in clinical practice.

From this editorial perspective, AI methods should be considered as members of a multidisciplinary team, providing explanations for their decisions and allowing clinicians to provide feedback in a constructive manner, centered on the unique needs of each patient.

Nonetheless, new guidelines for data protection and emerging ethical considerations regarding the use of AI should also be taken into account. These include the possibility of predicting an unfavorable prognosis before it becomes clinically evident, suggesting drugs that are not currently approved for a given indication, or revealing personal behaviors associated with a disease that patients may not wish to disclose or change. Although these ethical challenges are not new, AI may substantially increase the ability to infer and access such information, raising important questions about how these predictions and recommendations should be communicated, interpreted, and incorporated into clinical practice. Establishing appropriate ethical frameworks will therefore be essential to ensure that the increasing predictive power of AI supports patient care without compromising patient autonomy, privacy, and trust.

## Future perspectives

The studies presented in this Research Topic highlight how artificial intelligence is increasingly shaping immunological research across imaging, molecular modelling, and biomedical text mining. A key next step will be the integration of these complementary data streams into large-scale, interoperable frameworks linking molecular, cellular, and clinical information (1). To this purpose, resources such as the IMMUNEMAP open data platform (2), the Human Immune Health Atlas (3) and the Immune Dictionary (4), will be pivotal to enable integrative analyses of the immune system dynamics. To make this shift happen, from a technological point of view standardization of data formats is required, whereas data collection should be representative of diverse populations to avoid bias. Altogether, the availability of data encompassing FAIR principles will maximize reusage and the combination with AI-based algorithms ultimately supports a more unified and mechanistic understanding of immune-mediated disorders.
